# The enchanted loom of artificial general intelligence

**DOI:** 10.1093/nsr/nwag040

**Published:** 2026-01-30

**Authors:** Mu-ming Poo

**Affiliations:** Executive Editor-in-Chief of NSR

## Abstract

With this enchanted loom in the horizon, serious deliberation of what AGI entails and how it could impact society is in order.

The year 2025 witnessed unprecedented development of artificial intelligence (AI), from large language models capable of reasoning to AI agents for performing specific tasks and robots with some embodied intelligence. The coming year promises to be even more exhilarating. A full-blown race has begun among frontier labs and tech companies to build models towards artificial general intelligence (AGI), with generalizable cognitive capability comparable to, or even surpassing, human intelligence. With this ‘enchanted loom’ on the horizon, serious deliberation of what AGI entails and how it could impact society is in order.

AGI is supposed to exhibit all aspects of human intelligence. Two categories of intelligence could be broadly defined. First, the cognitive/analytical intelligence for interactions with the external world, such as sensory perception, multi-sensory integration, sensorimotor transformation, learning and memory, attention, reasoning, decision-making and language, many of which have been achieved to varying degrees by the current AI systems. In particular, large language models (LLMs) have surprisingly gained the capability of human language. This was previously considered to be human-unique intelligence for understanding and articulating an almost unlimited string of symbols with syntax and grammar. The classic Turing test of human language intelligence has largely been passed by LLMs in the past 3 years. Nevertheless, the capability of recursive self-improvement and selective long-term memory characteristic of human intelligence remains to be attained.

Largely missing in the existing AI systems is the second category of emotional/social intelligence. This involves the intelligence manifested in emotion and feeling, empathy, theory of mind, social behaviors, self-consciousness and purposefulness (or ‘free will’). Notably, these forms of intelligence could only be fully demonstrated when embodied in physical systems, such as humanoid robots that are capable of appropriate interactions with the external world. As intelligent robots are becoming a prominent area of AI development, there is an urgent need to rigorously examine whether and how emotional/social intelligence exists in AI systems and could be aligned with human needs. A reasonable approach is to inquire whether various experimental tests for assessing animal and human emotional/social intelligence could reveal analogous phenomena in embodied AI systems. This calls for comparative studies of natural and machine intelligence, with the aim of monitoring the emergence of AGI and setting appropriate guardrails and governance.

A case in point is empathy, a key aspect of emotional intelligence that provides the basis for social interactions crucial for the harmonious existence of social groups and survival of the species. Lack of empathy is associated with cognitive deficits in many human psychiatric diseases, and tools of assessment and intervention have been devised in clinical neuroscience. In developing humanoid robots for medical and family care purposes, for example, it is critical to monitor and install empathy, even at an enhanced level, in these robots, making them safer and more suitable for tasks they are designed for.

Whether AI systems could have consciousness is frequently debated. Studies of consciousness in humans and non-human primates have defined various aspects of consciousness, including conscious perception, states of consciousness, self-consciousness and metacognition (i.e. self-awareness of cognitive states), all of which could now be experimentally examined. Given that these aspects of consciousness represent emergent phenomena associated with the evolution of increasingly complex neural circuits in animals, consciousness-like behaviors are likely to progressively emerge in AI systems comprising increasingly complex artificial neural networks. Monitoring and setting guardrails for the emergence of self-consciousness and ‘free will’ are particularly important for developing human-aligned AI systems.

The pioneering neurophysiologist Charles Sherrington coined the phrase ‘enchanted loom’ in his 1942 book *Man on His Nature* to describe his vision of what happens in the cerebral cortex when the intelligent mind emerges in the human brain during arousal from sleep, when ‘…the head mass becomes an enchanted loom where millions of flashing shuttles weave a dissolving pattern, always a meaningful pattern though never an abiding one; a shifting harmony of subpatterns’. Since the mechanisms underlying the emergence of human intelligence remain to be fully deciphered, we may wonder whether the development of AGI could shed light on the mystery of the human mind. In the meantime, we should ponder how machines with embodied AGI could co-exist harmoniously and synergistically with humans and, more importantly, how machine AGI could affect the development, functioning and evolution of human intelligence.

